# 

**Published:** 1861-04

**Authors:** 


### WORKS PUBLISHED DURING THE QUARTER.

### 1. *Anatomy and Physiology.*

Surgical Anatomy of the Arteries, with the Descriptive Anatomy of the Heart. By John Hatch Power, M.D., F.R.C.S.I. With Illustrations from drawings by B. Wills Richardson, F.R.C.S.I. Fcap. 8vo, price 10s.

Ellis's Demonstrations of Anatomy. A Guide to the Knowledge of the Human Body by Dissection. Fifth edition, with 130 Illustrations on Wood, small 8vo, 12s. 6d.

Human Osteology: comprising a Description of the Bones, with Delineations of the Attachments of the Muscles, &c.; the General and Microscopic Structure of Bone and its Development; to which is added a Brief Notice of the Unity of Type in the construction of the Vertebrate Skeleton. By Luther Holden, F.R.C.S. Third Edition, 8vo, cloth, 16s.

How to Work with the Microscope. Illustrated with 32 plates, containing upwards of 160 figures. By Lionel S. Beale, M.B., F.R.S. Post 8vo, 5s. 6d.

Diagrams of the Nerves of the Human Body, exhibiting their Origin, Divisions, and Connexions, with their Distribution to the various Regions of the Cutaneous Surface, and to all the Muscles. By William Henry Flower, F.R.C.S. Folio, plates, 14s.

The Anatomist's Vade-Mecum; a System of Human Anatomy. By Erasmus Wilson, F.R.S. Eighth Edition revised, with 280 Engravings on Wood, fcap. 8vo, 12s. 6d.

Compendium of Human Histology. By C. Morel. Translated and edited by W. H. Van Buren. Royal 8vo, cloth, 14s.

### 2. *Medicine.*

The Successful Application of Charcoal Air-Filters to the Ventilation and Disinfection of Sewers. A Letter to the Right Hon. the Lord Mayor. By John Stenhouse, LL.D., F.R.S. 8vo, 6d.

The Medical Directory for 1861, containing in one volume the Directories for London and the Provinces, Scotland and Ireland. 8vo, 10s. 6d.

Foundation for a New Theory and Practice of Medicine. By Thomas Inman, M.D. London, M.R.C.P. Second edition, much enlarged, crown 8vo, cloth, 10s.

The Scale of Medicines with which Merchant Vessels are to be furnished, by Command of the Privy Council for Trade; Observations on the Means of Preserving the Health of Seamen; with Directions for the Use of the Medicines, and for the Treatment of various Accidents and Diseases. By T. Spencer Wells, F.R.C.S. Second Edition, fcap. 8vo, 3s. 6d.

The Forms, Complications, Causes, Prevention, and Treatment of Consumption and Bronchitis; comprising also the Causes and Prevention of Scrofula. By James Copland, M.D., F.R.S. 8vo, price 12s. 6d. cloth.

On Stammering and Stuttering; their Nature and Treatment. By James Hunt, Ph.D. Post 8vo, 3s. 6d.

Adulterations Detected; or Plain Instructions for the Discovery of Frauds in Food and Medicine. By A. H. Hassall, M.D. London. Second Edition, crown 8vo, woodcuts, 17s. 6d.
Constipated Bowels: the Various Causes and the Rational Means of Cure. By S. B. Birch, M.D. Post 8vo, 2s. 6d.
Sore-Throat, its Nature, Varieties, and Treatment; including the Use of the Laryngoscope as an Aid to Diagnosis. By M. Prosser James, M.D. Post 8vo, 4s. 6d.
Clinical and Pathological Notes on Pericarditis. By W. T. Gairdner, M.D., F.R.C.P. Edin. 8vo, 1s.
Hints on Insanity. By John Millar, M.R.C.P. Fcap. 8vo, 3s. 6d.
Suggestions on the Construction of Asylums for the Insane. Illustrated by a Series of Plans. By William Dean Fairless, M.D. 8vo, 1s. 6d.
Successful Treatment of Influenza, Sore Throat, Bronchitis, Asthma, Pneumonia, &c. By H. Goodday, M.R.C.S. Fcap. 8vo, 2s. 6d.
Medical Handbook; comprehending such Information on Medical and Sanitary Subjects as is desirable in Educated Persons. With Hints to Clergymen and Visitors of the Poor. By F. W. Headland, M.D. Fcap. 8vo, 5s.
The Medical Register, published under the direction of the General Council of Medical Education, and Registration of the United Kingdom, pursuant to an Act to Regulate the Qualifications of Practitioners in Medicine and Surgery. Royal 8vo, 4s.
A Treatise on Fever. By Robert D. Lyons, K.C.C., M.B.T.C.D. 8vo, 12s. 6d.
On Insufficiency of the Aortic Valves, in connexion with Sudden Death; with Notes Historical and Critical. By John Cockle, M.D., F.L.S. 8vo, sewed, 1s.
The Lunacy Laws: a Letter to the Right Hon. the Earl of Shaftesbury. By P. R. Nesbitt, M.D. 8vo, price 6d.
The History of Medicine: comprising a Narrative of its Progress from the Earliest Ages to the Present Time, and of the Delusions incidental to its advance from Empiricism to the dignity of a Science. By Edward Meryon, M.D., F.G.S. Volume 1, 8vo, 12s. 6d.
Principles of Forensic Medicine. Second Edition rewritten. Illustrated by Wood Engravings. By W. A. Guy, M.B. Cantab., &c. 8vo, 10s. 6d..
Nature in the Cure of Disease. A Lecture by John M. Strachan, M.D. Crown 8vo, sewed, 6d.
A Practical Treatise on Diseases of the Skin in Children. From the French of Caillault. With Notes. By Robert Howarth Blake, M.R.C.S.L. Post 8vo, cloth, 9s. 6d.
Imperfect Digestion: its Causes and Treatment. By Arthur Leared, M.D., Member of the Royal College of Physicians. Second Edition, fcap. 8vo. 3s. 6d.
On Urine, Urinary Deposits, and Calculi: their Microscopical and Chemical Examination. Including the Chemical and Microscopical Apparatus required, and Tables for the Practical Examination of the Urine in Health and Disease; the Anatomy and Physiology of the Kidney; with original Analyses of the Urine in Disease, and General Remarks on the Treatment of certain Urinary Diseases. Illustrated with numerous Engravings. By Lionel S. Beale, M.B., F.R.S. Post 8vo, cloth, 8s. 6d.
The Eastern, or Turkish Bath: its History, Revival in Britain, and Application to the Purposes of Health. By Erasmus Wilson, F.R.S. Fcap. 8vo, 2s.
Diphtheria: its Symptoms and Treatment. By William Jenner, M.D. Fcap. 8vo, 2s. 6d.
On Food: being a Course of Lectures delivered at the South Kensington Museum. By E. Lankester. First Course. Sewed, 1s.

The Medical Missionary in China: a Narrative of Twenty Years' Experience. By William Lockhart. Cloth, 15s.

### 3. *Surgery.*

Clinical Surgery, Part II. The Surgical Diseases and Injuries of the Nose, Larynx, Thorax with its contents, and of the Organs of Circulation. By Thomas Bryant, F.R.C.S. 3s. 6d.

A Treatise on Diseases of the Joints. By Richard Barwell, F.R.C.S. With Engravings on Wood, 8vo, 12s.

A System of Surgery, Theoretical and Practical, in Treatises by various Authors, arranged and edited by T. Holmes, M.A. Cantab., Assistant-Surgeon to the Hospital for Sick Children. Volume one—General Pathology. 8vo, 1*l.* 1s.

Practical Observations on the Diseases of the Joints involving Anchylosis, and on the Treatment for the Restoration of Motion. By Bernard E. Brodhurst, F.R.C.S. Third Edition, enlarged 8vo, 4s. 6d.

On Diseases and Deformities of the Spine, Chest, and Limbs. Part I., Diseases of the Spine, causing Posterior Angular Projection, Abscess and Paralysis. By Richard Hughes, M.R.C.S., L.R.C.P. Edin. 8vo, price 1s. 6d.

On Gonorrhœa and Gleet. By T. C. Weedon Cooke, M.R.C.S. 8vo, 1s.

An Account of Two New Methods of Treating Aneurisms. By William Colles, A.B., T.C.D. 8vo. *Printed for private circulation.*

A Practical Treatise on the Ætiology, Pathology, and Treatment of the Congenital Malformations of the Rectum and Anus. By William Bodenhamer, M.D. Illustrated by 16 Plates, and exemplified by 287 Cases. 8vo (New York), 12s.

### 4. *Chemistry.*

Fownes's Manual of Chemistry. Edited by H. Bence Jones, M.D., F.R.S. and A. W. Hofmann, Ph. D., F.R.S. Eighth Edition, revised, fcap. 8vo, 12s. 6d.

A Manual of Photographic Chemistry, including the Practice of the Collodion Process. By T. Frederick Hardwich. Sixth Edition, revised, fcap. 8vo, 7s. 6d.

A Manual of Qualitative Analysis. By Robert Galloway, F.C.S. Third Edition, post 8vo, 5s.

### 5. *Natural Sciences.*

John Hunter's Essays and Observations on Natural History, Anatomy, Physiology, Psychology, and Geology: being his Posthumous Papers on those Subjects; arranged and revised with Notes. To which are added, the Introductory Lectures on the Hunterian Collection of Fossil Remains, delivered in 1855, by R. Owen, F.R.S. 2 vols. 8vo, 31s. 6d.

Memoir on the Megatherium; or, Giant Ground-Sloth of America (Megatherium Americanum, Cuvier). By Richard Owen. 4to, with 27 Plates, 42s.

